# Clinical interpretation of copy number variants in the human genome

**DOI:** 10.1007/s13353-017-0407-4

**Published:** 2017-09-30

**Authors:** Beata Nowakowska

**Affiliations:** Department of Medical Genetics, Institute of Mother and Child, Kasprzaka 17a, 01-211 Warsaw, Poland

**Keywords:** Copy number variants, CNV interpretation, VOUS, Susceptibility loci, Genotype–phenotype correlations

## Abstract

Molecular methods, by which copy number variants (CNVs) detection is available, have been gradually introduced into routine diagnostics over the last 15 years. Despite this, some CNVs continue to be a huge challenge when it comes to clinical interpretation. CNVs are an important source of normal and pathogenic variants, but, in many cases, their impact on human health depends on factors that are not yet known. Therefore, perception of their clinical consequences can change over time, as our knowledge grows. This review summarises guidelines that facilitate correct classification of identified changes and discusses difficulties with the interpretation of rare, small CNVs.

## Introduction

As cytogenetic and molecular technologies have developed, cytogenetically visible segments (Jacobs et al. 1992) and single-nucleotide polymorphisms (SNPs) (Lander et al. 2001; International HapMap Consortium 2005) have been described. But for over the past decade, researchers revealed that our genome contains multiple regions of intermediate size copy number changes, gains and losses, termed copy number variants (CNVs) (Iafrate et al. 2004; Sebat et al. 2004). These variants can range in size from several dozens of bases (> 50 bp) (MacDonald et al. 2014; Zarrei et al. 2015) to megabases and, within a single human genome, can result in 1.2% difference compared to the reference human genome (Pang et al. 2010). In 2006, Redon et al. constructed the first CNV map of the human genome, through the investigation of 270 apparently healthy individuals from four populations with ancestry in Europe, Africa or Asia (Redon et al. 2006). Using the Whole Genome Tile Path array (WGTP), which comprised of 26,574 large insert clones representing 93.7% of the euchromatic portion of the human genome (Fiegler et al. 2006), the average number of CNVs detected per genome was 70 and the mean size was 341 kb (Redon et al. 2006). Recently, an updated, higher resolution map of CNVs that are not associated with adverse phenotypes, based on 55 studies, was developed (Zarrei et al. 2015). Zarrei et al. estimated that up to 9.5% of the genome contributes to CNV. Additionally, they have found approximately 100 genes that can be homozygously deleted without producing apparent phenotypic consequences. This map is a great contribution to the interpretation of new CNV findings, for clinical and research applications (Zarrei et al. 2015). But CNVs are an important and large source of both normal and pathogenic variants, and the major challenge associated with CNVs is the estimation of whether the variation is benign or affects vital biological function and results in disease. It can be especially difficult with rare and non-recurrent variants, because of their extensive spectrum of effects, from lethality to adaptive features. Additionally, the borderline where the association of the phenotype with the CNV starts can be very subjective and the resolve may change over the time. Furthermore, whether or not a given CNV is of clinical consequence may depend on the set of other factors, like ethnical background or environmental elements (Zarrei et al. 2015). One of the first associations between CNV and phenotype was shown in 1936, when Bridges and co-authors described *Drosophila melanogaster* with Bar eye phenotype caused by duplication of the Bar gene (Bridges 1936). CNVs can influence phenotype and cause disease directly by disrupting genes and/or altering gene dosage (Lupski et al. 1992; McCarroll et al. 2006; Redon et al. 2006). Furthermore, CNVs can impact gene expression indirectly through position effects (Freeman et al. 2006; Feuk et al. 2006), by unmasking recessive mutation or by altering communication between alleles by deleting regulatory elements (Mikhail 2014). Discussion was conducted as to whether the term “variant” can be used in the context of both pathogenic and benign changes, which is not consistent with the terminology used for classical cytogenetic and single-nucleotides mutations. However, our increasing awareness of the inconsistent associations between CNVs and phenotypes suggests to use the term “variant” without implications on pathogenicity, frequency or other characteristics (Iafrate et al. 2004; Sebat et al. 2004; Lee and Scherer 2010).

## Technological advances in the detection of CNVs

Large-scale CNVs were initially detected with conventional karyotyping in the early days of cytogenetics (Jacobs et al. 1992). The past 15 years have realised rapid development in the technology and analysis of the human genome. This created a new field of investigation that transformed the clinical practice. Two primary technologies for the detection of CNVs are array comparative genomic hybridisation (aCGH) and the recently introduced high-throughput sequencing.

### Arrays

Arrays measure the fluorescent ratio of two labelled DNA samples (Kallioniemi et al. 1992), which competitively bind to many probe sequences attached on an array. When the values significantly deviate from the baseline, it indicates loss or gain with respect to the reference genome. Initial CNV detection was with arrays having a resolution of ~1 Mb (Greshock et al. 2004), and later close to 50 kb (Fiegler et al. 2006). The current generation of genomic arrays contains even millions of probes, and the resolution can be as high as a single exon in genes of interest (Boone et al. 2010). But even smaller pathogenic CNVs can occur, and they are often beyond the resolution limit of all genomic arrays (Boone et al. 2010, 2013; Hehir-Kwa et al. 2015).

### Next-generation sequencing

The development of next-generation sequencing (NGS) technologies has enabled the sequencing of millions of reads in parallel. But, more importantly, it allows the detection of variants as small as single-nucleotide deletion/duplication and other forms of structural variants (Metzker 2010; Ng et al. 2010). Since the detection of small changes dramatically increased, the definition of CNVs has widened from 1 kb (Feuk et al. 2006) to much smaller events. Now, the size of CNVs are typically defined as larger than 50 bp (Alkan et al. 2011; Zarrei et al. 2015). Several new methods for structural variants analysis have been developed: (1) paired-end mapping, where the genomic DNA is fragmented and cloned into fosmids (Korbel et al. 2007; Kidd et al. 2008); (2) read-depth analysis, which investigates change in read coverage (Alkan et al. 2009); and (3) split-read strategy, to detect paired-reads where only one end is uniquely mapped onto a reference genome (Hehir-Kwa et al. 2015). All the described methods suffer from different limitations; however, they present complementary advantages, therefore, combining approaches can definitely empower the detection of CNVs (Mills et al. 2011; Valsesia et al. 2013).

Recent implementation of CNVs detection, additionally to single-nucleotide variants, using NGS may have the potential to reduce the number of genomic assays required for a patient to one test to reach the diagnosis. High-throughput sequencing is perceived as the final goal for genetic testing, but it will take at least the next few years to replace the arrays completely, because of its cost, accessibility, robustness and turnaround time (Hehir-Kwa et al. 2015). During these years, we need to build our knowledge and experience on how to interpret comprehensive genomic data.

## Clinical interpretation guidelines

For clinical use, every detected CNV must be interpreted (South and Brothman 2011; de Leeuw et al. 2012). Geneticists need to distinguish pathogenic or high-risk from benign variants. But it should be underlined that the interpretation of CNVs depends substantially on the clinical indications. Clinicians need to provide sufficiently detailed clinical phenotypes, to allow correct interpretation of the result (Vermeesch et al. 2012). Several groups developed a graphical workflow for the interpretation of CNVs, which is very useful in the daily diagnostics work (Gijsbers et al. 2011; Vermeesch et al. 2012). To classify CNVs, several pieces of information need to be considered.

### Parental inheritance

In many cases, particularly when the small, rare CNV has been detected, parental testing is necessary for full/better interpretation. Arrays can identify private familial variants, not previously observed in a cohort of patients nor in an apparently normal control group (Itsara et al. 2009; Mencarelli et al. 2008; Vermeesch et al. 2012). Additional familial samples (like siblings) may also be required to determine whether a particular CNV is segregating with the phenotype within a family (Vermeesch et al. 2012). However, there are many reports of inherited pathogenic CNVs that can be of variable expression/penetrance, so caution should be taken not to automatically classify such variants as benign (Buysse et al. 2009; Fernandez et al. 2010; Cooper et al. 2011; Kaminsky et al. 2011). In addition, inherited deletion can reveal a recessive disorder due to a mutation on the remaining allele in an affected child.

### Databases

The databases available for CNV interpretation can be divided into three main categories: (1) Database containing information on individuals with different clinical phenotypes. The first type of databases collect individual cases regarding genetic and phenotypic details. These are sources such as Online Mendelian Inheritance in Man (OMIM), human genome browsers (UCSC, Ensembl), DatabasE of Chromosomal Imbalance and Phenotype in Humans using Ensembl Resources (DECIPHER) (Firth et al. 2009), European Cytogeneticists Association Register of Unbalanced Chromosome Aberrations (ECARUCA) (Feenstra et al. 2006) and International Standards for Cytogenomic Arrays (ISCA) (Kaminsky et al. 2011). (2) A database containing information about population studies of healthy people. These databases are created by collating data from different sources and represents the characterisation of “healthy” individuals, providing more genetic information about the genomic region of interest (Lee and Scherer 2010; de Leeuw et al. 2012). An example of such a database is the Database of Genomic Variants (DGV), which was created in 2004 (Iafrate et al. 2004) as a comprehensive catalogue of human CNV and structural variation among “control” individuals, and the 1000 Genomes Project, which was the first to collect whole-genome sequencing data of multiple samples from many populations (1000 Genomes Project Consortium 2010). (3) An “in-house” dataset, which is created based on cases processed by the laboratory itself.

However, when using the public databases, several points need to be considered: (1) The use of different array platforms included in the public databases can lead to differences in the reported size of identical CNVs (Haraksingh et al. 2011). In particular, many of the benign CNVs reported earlier are based on bacterial artificial chromosome (BAC) microarray studies and may represent size overestimates (Perry et al. 2008). (2) Sex information about the included individual is not always given. This consideration is particularly important for X-linked CNVs in males, as many of the reported benign variants included in databases are seen in females. However, in men who have only one X chromosome, the same change may already be pathogenic. (3) The majority of CNVs reported from large population studies have not been validated. (4) Factors such as incomplete penetrance, variable expressivity, age of onset and parent of origin imprinting effects are not considered. (5) Many publications use the same reference set (e.g. HapMap); therefore, a CNV represented in multiple publications may represent the same individual studied multiple times (Kearney et al. 2011).

### Consideration of CNV size

The microscopically visible CNVs are almost always associated with phenotypic consequences. As the size decreases, the more genomic variants are of clear clinical effect (Buysse et al. 2009). Systematic assessment of the population frequency of CNVs at different size ranges showed a significant increase in large CNVs in the affected cohort compared to the control group (Cooper et al. 2011). Only 8% of the general population carries a CNV larger than 500 kb, in contrast to almost 25% of patients with intellectual disability (Itsara et al. 2009; Cooper et al. 2011; Coe et al. 2012). Although conclusions drawn between CNV size and its clinical significance is true as a general rule, it is clear that very large CNVs can be benign in nature (Barber 2005; Filges et al. 2009; Itsara et al. 2009; Bateman et al. 2010) and very small CNVs can be clinically important (Nowakowska et al. 2010).

### Consideration of genomic content

Interpretation of non-recurrent CNV should consider whether the CNV contains unique, gene-rich sequences or is comprised of repetitive elements or pseudogenes. The gene content should be carefully analysed for relevant clinical associations and for dosage sensitivity (Huang et al. 2010; Kearney et al. 2011). When the genes are reported as pathogenic in the medical literature, the nature of these variants should be carefully investigated, taking into account: (1) A gene associated with a clinical phenotype due to haploinsufficiency (or mutation) may have no phenotype associated with a copy number gain. (2) Disorders often result from gain of function mutations rather than dosage imbalance. Therefore, CNVs involving such genes may either have no clinical relevance or result in an entirely different phenotype (e.g. mutations in FGFR1 result in skeletal dysplasia, whereas deletions are associated with Kallmann syndrome) (Wilkie 2005). (3) Copy number gains involving only part of a gene may result in gene disruption or altered coding sequence (Swensen et al. 2009). (4) Deletion of genes associated with recessive diseases may suggest mutation on the second copy of the gene. (5) Small variants involving only intronic sequences may still have an effect on the gene function. However, when the genes within the detected CNV are not reported in the literature as pathogenic, and the role of the gene is based only on predicted gene function, or function characterised in model organisms, the conclusions about pathogenicity cannot be drawn until the variant is not well characterised in the human population.

## Classification of variants

Using the guidelines presented above for the systematic classification of a CNV, each CNV can be assigned to one of three main categories of clinical significance. These categories should be used in clinical reporting:

1. Benign variants: The CNV is not enriched in individuals with certain phenotypes, has been reported in multiple peer-reviewed publications and is repeatedly found in the normal population.
2. Pathogenic variants: The CNV is well documented as clinically significant in multiple peer-reviewed publications, even if penetrance and expressivity of the CNV are known to be variable.
3. Variants of uncertain significance (VOUS): Represent a broad category of CNVs which have not been reported or, if reported, insufficient evidence is available for its unambiguous clinical significance. All CNVs which cannot be classified as pathogenic or benign are included in this group. Three categories of VOUS are suggested: (a) *likely pathogenic*: the CNV was described previously in a single patient with a similar phenotype, or a gene within this CNV has a function relevant to the reason for the patient’s referral; (b) *likely benign*: CNV includes no genes but it exceeds a size criterion for reporting; (c) *VOUS with no sub-classification*: CNV contains genes, but it is not known whether the genes are dosage sensitive and little is known about their function (Vermeesch et al. 2012). However, what is important, is that, every CNV that is initially classified as a VOUS may be reclassified to either a benign or pathogenic category as experience and scientific knowledge about the CNV grows over time (Westerfield et al. 2014).

## Incomplete penetrance/variable expressivity/susceptibility loci

CNVs of variable expressivity/incomplete penetrance are pathogenic variants that cause a true challenge in counselling. These are genetic risk factors very often associated with variable phenotypes and are more likely to be inherited (Coe et al. 2012). A classic example of variable expressivity is 22q11.21 deletion syndrome, where traditional inheritance of a genetic variant is used as a definitive factor for pathogenicity. De novo aberrations are always thought to be more deleterious, whereas inherited rearrangements are generally considered benign. However, for some CNVs like distal 1q21.1 (Mefford et al. 2008), 16p11.2 microdeletions/microduplications (Marshall et al. 2008; Weiss et al. 2008; Rosenfeld et al. 2010; Walters et al. 2010; Girirajan et al. 2011; Jacquemont et al. 2011; Coe et al. 2012), 16p11.2 proximal duplication (Giaroli et al. 2014) or 15q13.3 (Sharp et al. 2008; Cooper et al. 2011; Kaminsky et al. 2011; Deutsch et al. 2016), despite variable phenotypes and inheritance from normal parents, enrichment among affected individuals, compared to a healthy population, implicated them as pathogenic variants (Rosenfeld et al. 2013). As increasing numbers of cases and controls are studied, many susceptibility loci have recently been discovered (Girirajan and Eichler 2010; Girirajan et al. 2011; Cooper et al. 2011; Kaminsky et al. 2011). Based on the report by Rosenfeld et al., the combined incidence of the most common susceptibility variants, in the control population, is approximately 1/125 (Rosenfeld et al. 2013). The phenotype resulting from such susceptibility CNVs is unpredictable. Identification of one of these susceptibility CNVs can explain part of the genetic aetiology of the disorder (Girirajan and Eichler 2010; Girirajan et al. 2011), but, theoretically, the pathogenicity of these CNVs can be influenced by the second “hit”, either genetic or environmental, like the presence of an additional CNV (Girirajan et al. 2012), mutation or ethnic background (Vanakker et al. 2014). Some CNVs are associated with a much higher risk than others for a severe phenotype, i.e. distal del1q21.1, distal dup1q21.1, proximal del1q21.1, distal del16p11.2, del16p11.2, del17q12 and dup22q11.2 (Vanakker et al. 2014), and, for those, the penetrance rate has been calculated. The CNVs with a larger difference between cases and controls, and those with higher de novo frequencies, have higher penetrance rates (Rosenfeld et al. 2013). For less common CNVs, screening a larger control group would be necessary to estimate their penetrance rate. But the calculation model can already be a useful tool in postnatal as well as prenatal genetic counselling, providing at least some information to future parents. It can help to put the estimation of risk into perspective; for example, counselling about a 15q11.2 deletion could be relatively reassuring with a ~10% chance of penetrance, as compared with a ~62% chance of an abnormal phenotype with a 16p11.2 proximal deletion. The additional factors that may affect the phenotype in the vast majority are not known, and even if the second CNV is identified, it is not possible to predict how the CNVs may interact (Rosenfeld et al. 2013).

## Rare CNVs in neurodevelopmental disorders

CNVs can occur at different frequencies in the population. When the frequency is lower than 1%, CNV is considered to be rare, in contrast to common or polymorphic CNVs, which occur in the population with frequency higher than 1% (Valsesia et al. 2013). Both types of CNVs can occur in a normal population as well as in patients with abnormal phenotypes (Redon et al. 2006; Valsesia et al. 2012). However, studies from the Wellcome Trust Case Control Consortium found that only very few common variants were associated with diseases (Wellcome Trust Case Control Consortium and Craddock 2010), because for rare CNVs, such an association study is much more difficult and requires a large cohort to obtain statistical power. Therefore, the association with disease can be especially challenging for rare CNVs. However, it has been already proven that these rare CNVs are particularly enriched in individuals with complex neurodevelopmental phenotypes (Coe et al. 2012; Iyer and Girirajan 2015).

Neurodevelopmental disorders, like intellectual disability (ID), epilepsy, autism or schizophrenia, are characterised by neurological and psychiatric features occurring during brain development, and often have very complex aetiology. Intellectual disability, as an example, affects up to 3% of the population and is extremely heterogeneous in its origin. In the OMIM database, over 1000 known genetic conditions have ID as a component of the phenotype, and over 50 syndromes associated only with the X chromosome (Grayton et al. 2012). Studies have shown that, in children with unexplained ID, developmental delay and congenital anomalies, 15–20% will have a pathogenic CNV identified by an array, compared to only ~3% analysed with karyotypes. The last decade has increased our understanding of the genetic aetiology of these common disorders, with growing evidence that rare variants play a special role in this group of patients (Sebat 2007; Grayton et al. 2012). Often, rare CNVs also contain multiple genes, and it has been challenging to identify single genes and correlate them with specific features of the phenotype. In the past, in many cases, gene discovery was aided by chromosomal translocations or inversions, disrupting causal genes. Examples of such events include the *NSD1* gene in Sotos syndrome (Kurotaki et al. 2002), *SHANK3* in Phelan–McDermid syndrome (Bonaglia et al. 2001) or *UBE3A* in Angelman syndrome (Kishino et al. 1997). But also, CNVs from patients revealing the same phenotype, with overlapping deletions or duplications, have been used for identifying the minimum critical region carrying the candidate gene (Nowakowska et al. 2010). This approach is limited, however, by the availability of patients with the same phenotype.

## Different impact models of CNVs

The association of genes, involved in rare CNVs, with phenotypes can be categorised under three models (Iyer and Girirajan 2015): (1) Single gene model, when there is one major gene that lead to the phenotype. An example is the *MEF2C* gene (Nowakowska et al. 2010). However, it has become evident that an individual CNV affecting even one gene can give rise to many different neuropsychiatric phenotypes. One of these is a neurexin-1 gene (*NRXN1*), located on the 2p16.3 chromosomal region. Deletion of this gene is associated with ID, developmental delay, autism and has also been reported in schizophrenia patients (Kirov et al. 2009; Wiśniowiecka-Kowalnik et al. 2010). In a case–control study, the SGENE+ Consortium tested the association for exonic CNVs in *NRXN1* in 2977 schizophrenia patients and 3746 controls from seven European populations. They found a significant association, 0.24% versus 0.015% in the case versus control groups (Rujescu et al. 2009). (2) Contiguous gene model, where many genes within the CNV contribute to the phenotype, which results in multiple unrelated features in a single individual. A typical example of this model is 22q11 deletion syndrome, seen in patients with ID, schizophrenia and many other clinical features. Despite its variable phenotype, and variable expressivity of most features, common characteristic features make the 22q11 deletion a defined syndrome (Biswas and Furniss 2016). Also, the 17q12 deletion has typical presentation but its psychiatric presentation is very variable (Moreno-De-Luca et al. 2010). These two syndromes have core features, so they can be recognised by clinical geneticists. But some CNVs have no obvious clinical findings. For example, deletions and reciprocal duplications of 16p13.1 reported in patients with ID and autism (Ullmann et al. 2007) or microdeletions and microduplications of 16p11.2 implicated in autism and schizophrenia (Weiss et al. 2008), 1q21.1 deletion, where some carriers have no obvious clinical findings and others have variable phenotype, which includes microcephaly (~50% of cases), ID (~30% of cases), seizures (~15%) and other malformations (Grayton et al. 2012). (3) The third model assumes the existence of genetic background and modifiers elsewhere in the genome, which can be illustrated by the 16p12.1 microdeletion (Girirajan and Eichler 2010). This model represents what is recently emerging, the evidence that multiple rare CNVs (de novo or inherited) may contribute to the genetics for conditions such as schizophrenia or autism, and likely to other medically important conditions (Hehir-Kwa et al. 2013). This creates situations of great complexity to analyse and interpret, and will continue to challenge medical researchers for years to come.

## CNVs in prenatal diagnostics

The implementation of arrays in prenatal diagnosis reflects the potential of this technique and fulfills the need for a diagnostic test with a higher resolution than conventional karyotyping. The majority of foetuses with abnormal ultrasound have normal karyotypes, but numerous reports have demonstrated an increased detection rate of clinically significant, submicroscopic genomic imbalances using aCGH (Wapner et al. 2012; Wapner and Levy 2014). The main difficulties and fears concern ethical problems due to variants of unknown significance and even more difficult is to counsel pathogenic variants of incomplete penetrance. Databases of both benign and pathogenic CNVs have been developed to help facilitate clinical interpretation. The difficulty arises when CNV was not previously seen or seen only rarely and its significance is unknown. The general consensus approach is not to report such variants, unless it comes with high suspicion of being pathogenic. It depends on several factors, discussed above. But most importantly, it depends on the clinical characteristics (ultrasound abnormalities) and positive family history (Vanakker et al. 2014). Fortunately, as databases and the literature continue to expand, findings of VOUS are less frequent. The initial interpretation of CNVs from the National Institute of Child Health and Development (NICHD) study found that VOUS occurred in approximately 2.5% of cases; when the same CNVs were reclassified 5 years later, only 1.5% remained uncertain (Wapner et al. 2012; Wapner and Levy 2014). Susceptibility CNVs are risk factors with reduced penetrance or variable expressivity, in which an identical genetic alteration can be associated with significant variation in the phenotype. Thus, the phenotype resulting from such CNVs is unpredicted. Again, the decision of reporting such a variant needs to be assessed in combination with ultrasound findings and family history (Vanakker et al. 2014). Theoretically, the pathogenicity can be influenced by the ethnic background or the presence of an additional variant somewhere else in the genome, then if the variant is inherited from a perfectly healthy parent, the risk is lower than in a family with clinical history (Vanakker et al. 2014). However, in most cases, it will remain impossible to predict whether the child will have a clinical manifestation. For this reason, the general recommendation is not to report such variants in prenatal cases (Vanakker et al. 2014). Nonetheless, this information found during prenatal testing can be of significant value in childhood management, allowing early intervention and treatment for affected children (Wapner and Levy 2014). Because of the high frequency of susceptibility CNVs, which was estimated to be 1 in every 500 cases, this group of CNVs is the most challenging. And in contrast to the VOUS, the number of susceptibility CNVs will instead grow with the new data. The guidelines on how to interpret and report results from prenatal array and how to counsel pregnant woman can be found in several publications, which also reflects the approaches in different countries (Rooryck et al. 2013; Vanakker et al. 2014).

## Conclusion

Recent studies demonstrated that copy number variants (CNVs) are widespread in our genome and play an important role in human genetic variation, accounting for both human population diversity and human genetic diseases. Although clinically relevant CNVs can explain abnormal phenotypes in up to 20% of individuals, interpreting the pathogenicity of CNVs remains challenging, and often relies on information about frequency from a healthy cohort and databases with previously reported CNVs. Many CNVs are considered benign, while others are clearly pathogenic. But between these two ends, a wide spectrum of variants can be identified. Additionally, the continuing evolution of genomic technologies for the detection of CNVs and the advent of the NGS projects means that thousands of small variants are expected to be found for a single individual in the near future. This will bring about even more problems regarding how to interpret and prioritise variants that might be potentially associated with disease (Valsesia et al. 2013). The only way to increase our knowledge and move forward is to share data between both clinical centres and population studies. The collection of genetic data available to a larger audience is growing fast. The most challenging part, however, remains obtaining and linking relevant clinical information to genetic observations in a structured way, to aid accurate data interpretation. Only by submitting and sharing data will the genetics community successfully search and interpret clinical data from patients with developmental disorders, with the aim of improving their healthcare worldwide (de Leeuw et al. 2012; Hehir-Kwa et al. 2013).
